# Brain Abnormalities in Individuals with a Desire for a Healthy Limb Amputation: Somatosensory, Motoric or Both? A Task-Based fMRI Verdict

**DOI:** 10.3390/brainsci11091248

**Published:** 2021-09-21

**Authors:** Martina Gandola, Laura Zapparoli, Gianluca Saetta, Carlo Reverberi, Gerardo Salvato, Silvia Amaryllis Claudia Squarza, Paola Invernizzi, Maurizio Sberna, Peter Brugger, Gabriella Bottini, Eraldo Paulesu

**Affiliations:** 1 Department of Brain and Behavioral Sciences, University of Pavia, 27100 Pavia, Italy; gerardo.salvato@unipv.it (G.S.); gabriella.bottini@unipv.it (G.B.); 2 NeuroMI-Milan Center for Neuroscience, 20126 Milan, Italy; carlo.reverberi@unimib.it; 3 Psychology Department, University of Milano-Bicocca, 20126 Milan, Italy; laura.zapparoli@unimib.it (L.Z.); paolainv81@gmail.com (P.I.); eraldo.paulesu@unimib.it (E.P.); 4 fMRI Unit, IRCCS Istituto Ortopedico Galeazzi, 20126 Milan, Italy; 5 Department of Psychology, University of Zurich, 8050 Zürich, Switzerland; gianluca.saetta@gmail.com; 6 Neuroradiology Department, ASST Grande Ospedale Metropolitano Niguarda, 20126 Milan, Italy; silvia.squarza@ospedaleniguarda.it (S.A.C.S.); maurizio.sberna@ospedaleniguarda.it (M.S.); 7 Department of Psychiatry, Psychotherapy and Psychosomatics, Zurich Psychiatric University Hospital, 8008 Zürich, Switzerland; peterbrugger.ch@gmail.com; 8 Neuropsychology Unit, Valens Rehabilitation Centre, 7317 Valens, Switzerland; 9 Cognitive Neuropsychology Centre, ASST Grande Ospedale Metropolitano Niguarda, 20126 Milan, Italy

**Keywords:** body integrity dysphoria, task-based fMRI, body representation, body awareness, secondary somatosensory cortex, somatosensory stimulation, motor task

## Abstract

Body integrity dysphoria (BID), a long-lasting desire for the amputation of physically healthy limbs, is associated with reduced fMRI resting-state functional connectivity of somatosensory cortices. Here, we used fMRI to evaluate whether these findings could be replicated and expanded using a task-based paradigm. We measured brain activations during somatosensory stimulation and motor tasks for each of the four limbs in ten individuals with a life-long desire for the amputation of the left leg and fourteen controls. For the left leg, BID individuals had reduced brain activation in the right superior parietal lobule for somatosensory stimulation and in the right paracentral lobule for the motor task, areas where we previously found reduced resting-state functional connectivity. In addition, for somatosensory stimulation only, we found a robust reduction in activation of somatosensory areas SII bilaterally, mostly regardless of the stimulated body part. Areas SII were regions of convergent activations for signals from all four limbs in controls to a significantly greater extent than in subjects with BID. We conclude that BID is associated with altered integration of somatosensory and, to a lesser extent, motor signals, involving limb-specific cortical maps and brain regions where the first integration of body-related signals is achieved through convergence.

## 1. Introduction

Body integrity dysphoria (BID, ICD-11 for mortality and morbidity statistics. Available online: https://icd.who.int/browse11/l-m/en, accessed on 16 September 2021—code 6C21) is an unusual disabling condition characterized by a long-lasting and compulsive desire for the amputation of physically healthy limb(s) (see review in [1,2]). As a consequence of this obsessive desire, individuals suffering from BID often refer to surgeons asking for an amputation. Attempts at self-amputation have also been reported [3]. A variant of this disease referred to as “paralyzation” (or “paralyzation variant”) in which subjects manifest the desire for non-functioning limbs has also been described [4,5,6]. Moreover, the desire for limb amputation is often associated with a particular behavior, called “pretending behavior” because the person with BID would pretend to be amputated by using different practices such as bending back or tying up a leg or by using aids such as crutches or wheelchairs [7].

This complex clinical spectrum suggests that this clinical condition is multifaceted.

BID was first classified as a paraphilia of purely functional psychological origin (i.e., apotemnophilia), somehow linked to a sexual disturbance [8,9]. In 2005, First [7] introduced the term “Body Integrity Identity Disorder” (BIID) to emphasize some similarities between this condition and the gender identity disorder [10]. First also suggested that BIID may be conceptualized as “an unusual dysfunction in the development of one’s fundamental sense of anatomical (body) identity” [7]. In 2011, McGeoch and co-workers [11] proposed that the desire for amputation arises from an early developmental distortion of the body image due to a right parietal dysfunction. They introduced the term “xenomelia” (from Greek: “xeno” = foreign and “melia” = limb) to better describe the hypothesized neurological etiology of this condition. Although BID was not included in the Diagnostic and Statistical Manual of Mental Disorders, Fifth Edition (DSM-5), it has been included in June 2018 in the International Classification of Diseases for Mortality and Morbidity Statistics, eleventh revision (ICD-11; https://icd.who.int/browse11/l-m/en, accessed on 16 September 2021) as body integrity dysphoria (BID; code 6C21). BID affects more frequently male subjects, with the majority of them desiring a left leg amputation and the desire of amputation more frequently concerning left-sided rather than right-sided limbs [6,7,10,11,12] (42% in [13,14]). However, there are also individuals with BID seeking for a bilateral amputation (30% in [13]) or a right-sided amputation (28% in [13]). 

### 1.1. Sensory Functions in BID

Recently, Stone and co-workers [15] demonstrated that individuals with BID have a normal perception of their legs showing a performance comparable with that of the controls in tasks measuring different aspects of body representation: visual perception of the lower limbs (i.e., template matching task: visually-guided judgments of length and width of distorted images of their own legs), tactile perception (i.e., tactile distance estimation task: judgments about the distance between two simultaneously applied tactile stimuli), and proprioceptive body representation (i.e., localization task: asking participants to localize unseen landmarks on their legs). The authors also demonstrated that individuals with BID perform normally in feet mental rotation suggesting the presence of a preserved ability in using the body representation of the affected part during the task [16].

### 1.2. Similarities and Differences between BID and Somatoparaphrenia

The clinical observation that the desire for amputation more frequently concerns the left side of the body reminds of somatoparaphrenia (SP), which is a delusional disorder concerning the body representation [17], generally associated with right hemispheric lesions, being the focus of these delusional symptoms the left limbs. These delusional symptoms are sometimes associated with aggressive attitudes towards the left paralyzed limbs: these are felt as non-belonging to the body by the patient. Some authors have suggested that the BID might also be grounded on a hardwired neurological malfunction rather than be of a pure “functional” psychological nature (e.g., see [11,12,18]). However, although SP and BID share some common clinical features, there are substantial differences between these conditions. First of all, the delusion of disownership, which mainly characterizes SP [19], is not present in individuals with BID, who are perfectly aware that the limb belongs to their own body, and they do not misattribute it to anyone else. Moreover, while SP has been more frequently described for the left upper hand [20], BID is more commonly reported for the lower left leg. Furthermore, a consistent difference is that SP is due to a macroscopic cerebral lesion more frequently of the right hemisphere, inducing paralysis of the limb that is not recognized (usually the left hand/arm). This paralysis seems to be an essential component to provoke the non-belonging feeling that characterizes SP. Moreover, BID is also described in rare cases for both legs simultaneously. This potential bilaterality of BID is a further important difference to SP, which always exclusively targets one side of a patient’s body. Finally, but more relevantly, while 63% of participants reported that the amputation “would correct a mismatch between the person’s anatomy and sense of his or her true self” [7], only 13% described a feeling of disownership for the affected limb (13%) [7].

### 1.3. A Neurological Origin for BID?

In recent years, a substantial body of evidence has been accumulated, suggesting a possible neurological origin of BID. The available studies can be divided into two main categories: (i) studies that focused on the registration of physiological parameters in such as skin conductance [21,22] that can prove abnormal autonomic response to sensory signals, and (ii) studies that investigated the presence of brain anatomical and/or physiological alterations using imaging techniques [11,14,23,24,25,26,27,28].

The first observation suggesting alterations of the physiological parameters in subjects with BID was obtained by Brang and co-workers [21] who found in two individuals suffering from BID heightened skin conductance response (SCR) to pinprick applied distal compared to proximal the desired line of amputation. The authors speculated that xenomelia might arise from a congenital right parietal dysfunction and of its connection with the insula, an important area for multisensory integration (i.e., mismatch between spared somatosensory processing and a “deficient higher-order representation” of the body in the superior parietal lobe, SPL). More recently, Romano and co-workers [22] found a reduced anticipatory response to stimuli approaching the limb affected by the desire for amputation and stronger SCR responses for stimuli contacting the non-accepted body part than those administered to the “not affected” body part. In both studies, the abnormal physiological responses were specific for the body part/parts affected by the desire of amputation.

To date, only a few studies investigated the neurofunctional basis of this condition using neuroimaging techniques (see Table 1). Three of them were interested in highlighting cerebral structural alterations in subjects with BID [14,23,24], one study used magnetoencephalography (MEG; [11]), and two studies used fMRI [26,27] during the execution of sensory, motor (i.e., tactile stimulation and movement of hands and feet [27]), or cognitive tasks [26]. Finally, two recent studies investigated the alterations in the structural [25] and functional brain connectivity [25,28], as well as in the gray matter density [28] in subjects with a desire for amputation. In what follows, because the methodology used for the present investigation is fMRI, we will describe in detail the findings of the neuroimaging studies published as of today. This will allow us to sketch a picture of what is currently known about the neurology of the condition and what questions still remain open.

### 1.4. Brain Morphometry and Resting State fMRI Studies

As illustrated in Table 1, the majority of the neuroimaging studies investigated the presence of structural abnormalities in the brain of individuals with BID. Hilti and co-workers [14] studied 13 subjects with a desire for an above-knee amputation (eight of the left leg, two of the right leg and three bilateral) and 13 controls. They found structural alterations (i.e., differences in cortical thickness and surface area), strongly lateralized to the right hemisphere, involving the right SPL and the inferior parietal lobe (IPL), the primary (SI) and secondary (SII) somatosensory cortex, and the anterior insular cortex in the right hemisphere. However, structural differences were also found in the left hemisphere in the IPL and SII (i.e., larger cortical surface area). The authors concluded that BID is the consequence of the malfunctioning of a distributed network involved in body ownership that include the majority of the brain regions well known to be part of the so-called “body matrix” [29]. Structural abnormalities were also found by a subsequent study by Bloom and co-workers [23] that demonstrated reduced grey matter volume in the left dorsal and ventral premotor cortices (PMCd and PMCv, respectively) and larger grey matter volume in the cerebellum (lobule VIIa) in a group of eight individuals with BID compared with 24 healthy subjects. Since both these regions are considered to be crucial for the feeling of body ownership and multisensory integrations, BID was interpreted by the authors as a dysfunctional integration of multisensory information [23].

The first evidence of structural alterations in somatotopically organized subcortical regions such as the basal ganglia and thalamus in subjects with BID was provided by Hängii and colleagues [24]. They found reduced grey matter in dorsomedial putamen bilaterally and a thickening (i.e., dilatation of shape) of both lateral and frontolateral of the thalami. The same authors, using DTI, fiber tractography, and rsfMRI, found structural and functional hyper-connectivity within the sensorimotor system in subjects with BID, mainly involving the right hemisphere. The regions included the SPL, SI, and SII, the premotor cortex, the basal ganglia, the thalamus, and the insula [25].

To summarize, these studies found structural alterations in subjects with BID in different regions including the superior (SPL) and inferior parietal lobe (IPL) [14,25], the insula [14,25], the primary (SI) and secondary (SII) somatosensory cortex (SII) [14,25], the paracentral lobule [25], the supplementary motor area (SMA) [25], the basal ganglia [24,25], the thalamus [24,25], the cerebellum [23,25], and the premotor cortex [23]. The majority of these brain regions are crucial parts of the distributed and interconnected neural system subserving the sense of awareness of one’s own body parts which relies on a distributed yet interconnected neural system rather than on a single specialized region [30]. In particular, the supramarginal gyrus [31], the temporoparietal cortex [32,33], the insular [34], and the premotor [32] cortices have been called into play in more than one study (see [35]). Moreover, SII, a somatotopically organized region of sensorimotor integration, may contribute to the online representation of the body (for an in-depth discussion of the role of this region in the generation of the sense of body ownership, see [36]).

In a resting-state fMRI brain connectivity and VBM study, recruiting so far the largest sample of BID individuals all desiring to have their left leg amputated (*n* = 16), we recently demonstrated that functional connectivity and grey matter density are altered in participants with BID. Interestingly, we found reduced intrinsic functional connectivity in the right dorsal paracentral lobule (rPCL) and superior parietal lobule (rSPL), and in the left inferior frontal gyrus (part orbitalis, lIFGOrb) and superior temporal gyrus (lSTG). Furthermore, we found lower grey matter density in the left ventral premotor cortex (lPCMv) and the rSPL in BID with a positive correlation between the grey matter density of the rSPL and both the desire of amputation and pretending behavior [28].

### 1.5. Task Based Functional Imaging Studies

Previous task-based fMRI or magnetoencephalography (MEG) studies on BID used tactile stimulation [11,27], motor tasks [27], or visual observation of virtually amputated bodies [26]. It is worth mentioning that these studies had extremely small samples and heterogeneous groups of participants with BID as far as the limb(s) and side of the desired amputation; four individuals with BID (two right leg, one left leg, one bilateral) in McGeoch et al. [11], five subjects (three right-leg BID, two left-leg BID) in van Dijk et al. [27] and 12 participants (nine left-leg BID, one right-leg, two both-legs BID) in Oddo-Sommerfeld et al. [26].

McGeoch and colleagues [11] recorded the somatosensory evoked fields in four individuals, using MEG scans, during tactile stimulation of the body parts above and below the desired amputation line. They found a significant reduction in the activity of the right superior parietal lobule (rSPL), when comparing somatosensory evoked responses for the affected leg with that of the unaffected leg, and that of controls. The authors interpreted this result by suggesting a conflict between preserved visual and sensory inputs concerning the affected limb and an “insufficient” representation of the same limb in the SPL: “sufferers would be able to see and feel a limb that nonetheless failed to incorporate into their body image” (p. 1317 in [11]). In 2013, van Dijk and colleagues [27] performed the first task-based fMRI study in BID using a tactile stimulation task (consisting of stroking the subjects’ skin with a brush), and a motor execution task of hands and feet (i.e., movement of the digits of one foot or hand alternated with rest in a classic block design). The study, performed in a small and heterogeneous group of subjects with BID (*n* = 5, three subjects with a desire for the amputation of the right leg and two for the left), revealed as the main effect a vast fronto-parietal area of increased activation, including somatosensory cortices, in response to dynamic brushes; on the other hand, they observed a group-by-leg interaction, a reduced activity in the left dorsal and ventral premotor cortex during the tactile stimulation of the “affected” (alienated) leg as compared with the “unaffected” (owned) leg. In contrast, no differences were found in the motor execution task. More recently, Oddo-Sommerfeld and colleagues [26] performed multivariate statistics using machine learning on imaging data collected while subjects viewed pictures of their own real and virtually amputated body. They found that brain activity recorded when viewing the picture of one’s own virtually amputated body predicted group membership with accuracy, sensitivity, and specificity higher than 80%. The more predictive brain regions were the right SPL and IPL, the caudate nucleus, and occipital areas.

### 1.6. Summary of Imaging Studies and Outstanding Issues

The observation of structural and functional anomalies in brain regions involved in the high-level body representation has led several authors to hypothesize a mismatch between preserved sensory processing and altered higher-level body representation to be characteristic of BID. However, most of these interpretations are based on forms of reverse inference whereby the putative deficit in high-level body representation is inferred on the basis of the “nature” of brain regions found to be less active or less densely packed of neurons. In principle, a more explicit demonstration of this concept and the underlying mechanism would be desirable. Below, we present our strategy to start pursuing this goal.

Furthermore, there is one other aspect that may deserve further investigation: body awareness is not only based on a somatosensory perception of the outside world through tactile/thermal/proprioceptive information, it also relies on motor signals that integrate body awareness both in terms of monitoring the status of the motor system or in terms of the individual contribution to the generation of consequences in the outside world through motor acts for what is normally defined as a sense of agency. Both aspects can be selectively deranged [37] and may be associated with delusional symptoms in acquired brain damage [38] or in developmental disorders [39].

The only fMRI activation study where a motor task was used [27] could not find any systematic difference between subjects with BID and healthy controls. However, the sample size in that study was small (*n* = 5) and the limb and side for which there was a desire of amputation varied from subject to subject, making the emergence of a systematic difference less than likely.

### 1.7. Aims of the Present Study

As pointed out, one of the main limitations of the previous literature, at least for our quest, was the small samples size and their heterogeneity together with the lack of a systematic investigation of both somatosensory and motor brain responses. In addition, there is no consensus in the literature on whether the brain response for somatosensory stimulation should be reduced or increased. As also outlined above, there is another important limitation to the interpretations on BID given so far. It is a fact that the purportedly high-level somatosensory body integration deficit, appears based primarily on reverse inference.

To overcome these limitations and shed new light on the neural correlates of the BID syndrome, we used fMRI to assess the functional brain organization of ten subjects who specifically shared the desire for the amputation of their left leg during movements and tactile stimulation of their hands and feet. Although this sample size is not dramatically larger than in previous studies, at the very least, it is twice as big as some previous investigations and fully homogeneous in terms of the body segment targeted by the desire for amputation (see also the section, “Limitations of the study” at the end of the discussion).

To try and test the nature of any neurofunctional deficit, we mapped brain regions that have neurons responding to a similar extent, to stimulation of each of the four limbs and to those that are active no matter which limb segment is moved. We reasoned that one of such regions should represent a building block in the making of an overall body representation, contributing to the integration of signals coming from single limbs into a more general and less segregated mapping of the body.

To further extend our investigation, we examined the somatosensory and motor brain systems for both upper and lower limbs of both sides. This allowed us to compare BID and healthy controls with the additional intra-subject reference point of the brain activation for the “unaffected” limbs.

### 1.8. Predictions

We had specific predictions in mind at the behavioral and functional–anatomical level, in line with different alternative scenarios that we shall call (A) “Elementary somatosensory”, (B) “Higher-order” somatosensory, (C) “Sensorimotor integration”, and (D) Purely motor.

According to an **Elementary somatosensory scenario A**, one should be able to demonstrate elementary somatosensory deficits at a clinical examination calling into play a deficit in the response of fine-grained somatotopically organized somatosensory cortex, for example, area S1. Scenario A is compatible with a downstream involvement of other somatosensory areas (e.g., area SII) with broader mapping at individual neurons of the body surface, as documented in studies with natural [40] or experimental [41] lesions of area S1. All group-specific differences should emerge from a somatosensory stimulation task.

According to the **Higher-order somatosensory scenario B**, one should expect to replicate the finding that subjects with BID have a grossly normal clinical examination of elementary somatosensory functions together with the normal activation of somatotopically organized somatosensory cortices such as area S1; it would remain contentious whether one could detect abnormal responses in brain regions that have a broad mapping of the body and responding for stimuli from both sides of the body and both limbs: the first brain region that shows such properties is area SII (reviewed in [42]). Most group-specific differences should emerge from a somatosensory stimulation task.

With the **Sensorimotor integration, scenario C**, besides abnormal responses in high-order somatosensory cortices, one should expect functional abnormalities also within the motor system, in regions involved in motor awareness such as the ventral premotor, or the pre-SMA, associated with motor monitoring [37] and the sense of agency [43] rather than in area M1: group-specific differences should emerge for both a somatosensory stimulation and for a motor task.

The **Purely-motor scenario D** entertains the, for the time being, highly unlikely hypothesis that abnormalities might emerge only within the motor system, that is within regions whose primary functional emphasis is, even though not exclusively, motoric in nature (e.g., area M1 or premotor cortex): the predictions of scenario D would be fulfilled providing that the group-specific differences would emerge primarily in a motor task. No evidence has ever been provided for such a restrictive scenario that we considered for theoretical reasons.

At the outset of our study, we favored scenario C with a weaker second preference for scenario B. However, whatever the outcome of our experiments, what counted to us was that our experimental design and analytical techniques allowed us to test the different outcomes of the various scenarios, without being biased by our initial expectations, as we described next. Besides considering the group/limb specific differences as target outcomes, we mapped to what degree our sensory stimulations or motor performances converged in the activation of the same area(s): such conjunctions and the group specific differences thereof would point more explicitly to an aberrant integration of body signals into a general body schema for individuals with BID.

## 2. Materials and Methods

### 2.1. Participants

Subjects with BID were recruited either via online advertising on the Internet fora or during a conference about this disorder and by participants’ word-of-mouth. They were referred to the Cognitive Neuropsychology Centre of the ASST Grande Ospedale Metropolitano Niguarda of Milan after being recruited by P.B. and co-workers. All subjects with BID reported a lifelong and persistent desire to have an amputation of a functionally healthy body part, usually starting in childhood or adolescence. All subjects were able to exactly indicate the site of the desired amputation.

The present sample includes 10 male subjects all suffering from a long-lasting desire for the amputation of the left leg just above the knee (mean age: 41.50 ± 8.85 years; range: 34–64 years; mean education: 15.10 ± 2.51 years; range 13–18) and 14 healthy subjects (mean age: 38 ± 9.20 years; range: 26–53 years; mean education: 15.50 ± 3,98 years; range 8–18 years, see Table 2).

This study thus presents data from the largest homogeneous sample presented in the literature to date using an activation fMRI protocol. Eight of the 10 BID participants included in the present study and all control subjects also took part in the grey matter density and functional connectivity study by Saetta and co-workers [28].

Informed consent was obtained from all subjects involved in the study and the study was approved by the Local Ethics Committee of the ASL of Milan (Comitato Etico Azienda Sanitaria Locale Città di Milano). The experiment was performed in accordance with the ethical standards laid down in the Declaration of Helsinki (1964).

### 2.2. Clinical Interview

All subjects completed a semi-structured qualitative clinical interview aimed to collect the following information: personal data (i.e., age, education, civil status, sport, and hobbies, etc.), degree of knowledge of family members and friends about the disease (e.g., does your partner/parents/kids/friends know about your desire?), treatments (e.g., medication, psychotherapy), assumption of drug/alcohols, smoking, onset of the desire (e.g., when was the first time that you felt the desire to be an amputee?), intensity and characteristics of the desire; motivation about the amputation and potential contacts with a surgical team in view of its realization; emotions connected with possible surgery; the way in which they had collected information about BID.

All the subjects with BID referred considerable distress, spent several hours per day thinking about the amputation and collecting BID and amputation-related information via the Internet. They described this condition as always present since childhood (“BID was always there”) however they had not yet contacted surgeons for amputation, nor had tried to perform it by themselves, still hoping to find an alternative solution for what they considered a neurological condition. As emerged from the participants’ clinical interviews, all these individuals fully satisfied the diagnostic criteria proposed for the BID diagnosis in the ICD 11 (https://icd.who.int/browse11/l-m/en, code 6C21, accessed on 16 September 2021).

### 2.3. Neurological and Psychiatric Assessment

The neurological evaluation was based on a basic assessment of motor function—somatosensory and visual perception for unilateral and bilateral stimuli [44]. The psychiatric assessment was performed through a clinical interview supervised by PB.

### 2.4. Zurich Xenomelia Scale

The desire for limb amputation has been systematically investigated by means of the Zurich Xenomelia Scale (English translation; [45]). The questionnaire contains 12 statements for which participants had to quantify their level of agreement, from total agreement (1 = strongly agree) to total disagreement (6 = strongly disagree), according to a Likert scale (see Appendix 1, page 110 in Aoyama et al., for the complete list of the items). The questionnaire includes three subscales that investigated different facets of the disorders: “pure amputation desire” (i.e., the strength of the amputation desire) items 1, 2, 5, 10, “erotic attraction” by amputees, items 3, 6, 9, 12, and “pretending behavior” (i.e., the extent to which the subject goes to pretend to be amputated), items 4, 7, 8, 11. In Table 2, the mean scores on each of the three subscales for each participant with BID are reported (see Table 2).

### 2.5. FMRI Experiment

The fMRI experiment involved a (i) hand and foot tactile stimulation task (non-intentional task) and a (ii) hand and foot motor execution task (intentional task).

#### 2.5.1. Tactile Stimulation Task

In the tactile stimulation task, the experimenter administered gentle manual stroking of the left or right finger or toes of the subjects according to the instructions received by means of MR-compatible earphones. The four conditions (right hand—RH, left hand—LH, right foot—RF, left foot—LF) were alternated with resting scans according to a block counter-balanced design (rest-RH; rest-LF; rest-RF; rest-LH; rest-LH; rest-RF; rest-LF; rest-RH; rest-RH; rest-LF; rest-RF; rest-LH). During the rest baseline conditions, subjects were instructed to relax and to think of nothing.

#### 2.5.2. Hand Motor Execution Task

During the task, the participants were asked to perform movements of the right and left hands alternating with periods of rest. The movements, performed at a frequency of approximately 1 Hz, involved thumb-to-finger sequential opposition: thumb to index finger, thumb to middle finger, etc. Subjects practiced the thumb-to-finger opposition task briefly until the desired pace was reached before the scanning. The task was self-paced, but the subjects were reminded to keep performing the task every 6 s with an auditory instruction (“move the right hand” or “move the left hand”). The auditory instructions were delivered using the software Presentation^®^ (www.neurobs.com, accessed on 16 September 2021) via fMRI-compatible headphones. These conditions were alternated with resting-state scans according to a block design. During the rest baseline conditions, subjects were instructed to relax and to refrain from thinking systematically. As before, subjects were reminded to remain in a resting state (“Rest, don’t move”) by a verbal instruction once every 6 s. Each block was 30 s long (10 scans in each epoch). The experiment consisted of three blocks of right-hand motion (RH), three blocks of left-hand motion (LH), and three rest blocks (rest) for each hand in a counterbalanced order (rest-RH; rest-LH; rest-LH; rest-RH; rest-RH; rest-LH). One experimenter (MG) was in the scanner room to monitor that healthy control and BID performed the task at the desired rate. All subjects performed the exceedingly simple task as requested.

#### 2.5.3. Foot Motor Execution Task

The task and timing were the same as described for the “hand motor execution task” (Section 2.5.2) but the movement requested was a flexion and abduction of the toes.

### 2.6. fMRI Data Acquisition and Analyses

#### 2.6.1. fMRI Data Acquisition

Functional MRI scans were performed using a 1.5 T General Electric (GE) Signa HD-XT scanner, equipped with an Echo Planar Imaging (EPI) gradient-echo sequence (flip angle = 90°; TE = 60 ms, repetition time (TR) = 3000 ms, field of view (FOV) = 240 × 240 mm and matrix size = 64 × 64). The selected volume consisted of 35 contiguous, interleaved, axial images (thickness = 5 mm; interslice gap = 0 mm), co-planar with AC-PC line, and acquired each 3 sec. For the tactile stimulation task, we collected 240 complete brain volumes. For both the motor execution tasks (hand and foot M.E.), we collected 120 complete brain volumes. The first 10 volumes of each sequence (corresponding to task instructions) were discharged from the analysis. A high-resolution T1-weighted whole-brain high-resolution MRI anatomical scan was also acquired for each participant using a 3D-SPGR sequence (flip angle = 20°; TE = 2.92 ms, TR = 9.2 ms, acquisition matrices = 256 × 256, slice thickness = 1 mm; interslice gap = 0 mm and voxel size 1 × 1 × 1 mm^3^). The volumetric MRI scans included 154 slices acquired on oblique sections parallel to the AC-PC line to cover the entire brain volume.

#### 2.6.2. Preprocessing

After image reconstruction, raw-data visualization and conversion from DICOM to the NIFTI format were performed using the program dcm2nii implemented in the software MRIcron. All subsequent data analyses were performed in MATLAB version 8.1 (Math Works, Natick, MA, USA) using the Statistical Parametric Mapping software (SPM12, Wellcome Department of Imaging Neuroscience, London, UK). The images were primarily examined for motion artifacts and evident anatomical abnormalities and then manually centered at the anterior commissure.

Subsequently, the fMRI scans were first realigned to the first scan of each run to account for any movement during the experiment. The structural image (T1) of each subject was segmented and the resulting image was co-registered with the mean functional image obtained after realignment. The data were stereotactically normalized into an MNI template space to allow group data analyses. At this stage, the data matrix was interpolated to produce voxels of dimensions 2 × 2 × 2 mm^3^. The stereotactically normalized scans were smoothed with a Gaussian kernel of 10 × 10 × 10 mm^3^ to improve the signal-to-noise ratio [46,47].

#### 2.6.3. Statistical Analysis of the fMRI Data

The BOLD signal associated with each experimental condition was then analyzed by a convolution with a canonical hemodynamic response function [48]. The global differences in fMRI signals were removed by using proportional scaling for all of the voxels on the global counts. High-pass filtering (128 s) was used to remove artifactual contributions to the fMRI signal, such as physiological noise from cardiac and respiratory cycles.

##### First-Level Fixed-Effect Analysis

A fixed-effect block analysis was performed first in each subject to characterize the BOLD response associated with each task as opposed to its baseline condition (rest), before entering the relevant contrast images into a random effect analysis. The realignment parameters were entered into the design matrix to further remove artifacts due to movement. For the tactile stimulation task, the following four contrasts were obtained: (i) right hand > rest, (ii) left hand > rest, (iii) right foot > rest and (iv) left foot > rest. For the motor execution task, the contrasts (i) right hand > rest, (ii) left hand > rest, (iii) right foot > rest and (iv) left foot > rest were obtained.

##### Second Level Random-Effect Analysis

The analyses of the fMRI data were completed with second-level full factorial ANOVAs, conforming to a random effect analysis to allow a generalization to the population level of the statistical inferences [49,50]. Hence the data were analyzed according to a factorial design by looking at the main effects of Groups, Body part, and Side and their interactions for the sensory stimulation blocks and for the motor performance blocks separately. When justified by a priori hypotheses, we also explored simple effects (e.g., responses to touches in the right hand in healthy controls).

All reported results survive a whole-brain cluster-level family-wise error rate (FWER) correction for multiple comparisons (p_FWER_ < 0.05). The voxel-wise threshold applied to the statistical maps before the cluster-wise correction is *p* < 0.001 uncorrected, as recommended by Flandin and Friston [51]. For clusters significant at the *p* < 0.05 FWER-corrected level, we also report the other peaks at *p* < 0.001. The peaks that survive the statistical threshold *p* < 0.05 whole-brain FWER-corrected voxelwise (peak level) are reported in the tables. Accordingly, all results described were corrected for multiple comparisons, using state of the art approaches [51,52] (Contrary to the suggestions of Eklund et al. [51] and as remarked by Flandin and Friston [52], preliminary spatial smoothing of 10 × 10 × 10 mm combined with a voxel-level preliminary threshold *p* < 0.001, makes cluster level FWER correction valid under the Gaussian fields theory framework, the number of false positives being within acceptable family-wise error rates). For high-level interaction effects, we also considered regions reaching an uncorrected *p*-value.

We also considered ROI-oriented analyses using as ROIs the regions identified in a resting-state fMRI functional connectivity study and voxel-based morphometry (VBM) published by Saetta et al. [28]. These regions displayed a reduced intrinsic functional connectivity in BID compared with controls; a subset also showed a reduced grey matter density. The four functional connectivity ROI included the right paracentral lobule (rPCL), the right superior parietal lobule (rSPL), the pars orbitalis of the left inferior frontal gyrus (lIFGOrb), and the left superior temporal gyrus (lSTG) (In the original paper by Saetta et al. [28], this region was incorrectly labeled as the left inferior temporal cortex. This labeling error has now been rectified in a Correction notice. Current Biology 2021, Volume 31, Issue 16, 23 August 2021, Page 3702). The VBM regions included a left premotor region, the right superior parietal lobule (rSPL), and the pars orbitalis of the left inferior frontal gyrus. Inside these regions, we tested significant reductions of neural activity for the main effect and interactions observed in the principal whole-brain analysis.

##### Conjunction Analyses

Finally, we also tested for the presence of conjunction effects for the activation patterns defined by each individual limb in each of the two experiments. The rationale for this analysis was that the brain has somatosensory regions with neurons having a broad mapping of the body surface: such neurons are contained, for example, in area SII, with receptive fields spanning, in some cases, across the two sides of the body and both upper and lower limbs (see [42] for review). We reasoned that a more limited response showing a conjunction effect may testify to a lower degree of integration of somatosensory or motor signals of single limbs within a more general body map. For these analyses, we used the conjunction null hypothesis offered by SPM12: this enabled voxelwise inference that there was activation in condition 1 **AND** condition 2 **AND** condition 3, etc., similarly to a logical **AND** rule.

##### Comparison of Conjunction Maps across Groups

There is no way of comparing the conjunction analyses of two groups or conditions in terms of their statistical significance at a voxel level. However, it is possible to test the hypothesis that each voxel shows a conjunction effect more frequently in a population rather than in another one. To do so, for each subject we calculated first-level conjunction analyses for the somatosensory tasks and for the motor tasks. We used a lenient *p* < 0.05 threshold for these maps to minimize type II error. The ensuing SPM[t]maps were saved in a binary format, whereby a nominal value 1 was given to each voxel showing a conjunction effect, a coding equivalent to that given to brain lesions in voxel-wise lesion symptom mapping (VLSM) analyses. Hence, the binarized individual SPM-maps of these individual conjunctions were later entered into a VLSM analysis using the Liebermeister test [53] to test the hypothesis in each voxel that a different proportion of subjects of the two groups, controls and individuals with BID, presented with the aforementioned joined response irrespective of the stimulated limb.

## 3. Results

### 3.1. Demographic and Clinical Data

The age and education of the subjects with BID (median age = 38.5; median education = 13.5) and of the healthy controls (median age = 39; median education = 18) were not significantly different as shown by the non-parametric Mann–Whitney U-tests: age: U = 57; Z = −0.762; *p* = 0.446; education: U = 59.5; Z = −0.677; *p* = 0.499. Neurological and psychiatric examinations proved normal in all participants, including those with BID. The data of the Zurich Xenomelia scale are reported in Table 2.

### 3.2. fMRI Results

#### 3.2.1. Within Group Activations for Somatosensory Stimulation and Motor Tasks

The within-group assessments of the activation patterns for the somatosensory stimulation and motor execution tasks were consistent with those previously found in the literature. This demonstrates the quality of the present fMRI data (e.g., see [54,55]).

##### Somatosensory Stimulation Task

Regions activated during tactile stimulation of hands and feet are reported in Figure S1 and Table S1. In both controls and subjects with BID, the tactile stimulation of hands and feet activated the bilateral sensorimotor cortex with more medial activations for the stimulation of the feet and more lateral activations for the stimulation of the hands (Figure S2).

##### Motor Execution Task

Brain regions activated in the motor execution task are shown in Figure S3 and Table S2. In both controls and subjects with BID, the motor execution tasks activated a large fronto-parietal and cerebellar network with more medial activation for the movement of the feet and more lateral activation for the movement of the hands.

#### 3.2.2. Between Group Comparisons

##### Somatosensory Stimulation Task

**Figure 1:**
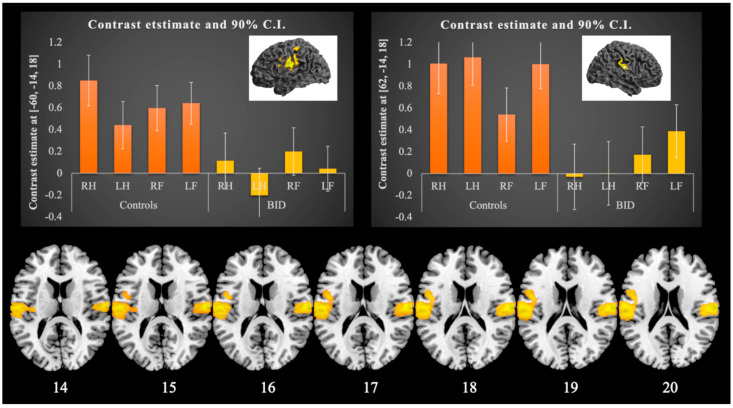
Between-group comparison: tactile stimulation task. Comparison between individuals with BID and healthy subjects. Brain regions more active in healthy subjects than in subjects with BID (linear t-contrast: healthy subjects vs. individuals with BID). The bar graphs (upper right and left graph) illustrate the contrast estimates—betas—and their 90% confidence intervals of the fMRI response in the left (MNI coordinate: x = −60; y = −14; z = 18) and right (MNI coordinate: x = 62; y = −14; z = 18) postcentral gyrus respectively: these regions showed a decreased activity in participants with BID during the tactile stimulation of the body parts. Visualized at the voxelwise threshold of *p* < 0.001 uncorrected.

The whole-brain full factorial fMRI analysis showed a main effect of Group, Body part, and Side. We found that subjects with BID showed a bilaterally reduced activation in the postcentral gyrus in a region corresponding to area SII and in the supramarginal gyrus (Figure 1, Table 3). This overall group difference survived a correction for multiple comparisons. Among the regions described by Saetta et al. [28], we found a significant group effect also in the left premotor cortex (x = −50; y = 2; z = 22; *p* = 0.00001, a peak in the context of the cluster #2 corrected for multiple comparisons, Table 3).

**Figure 2:**
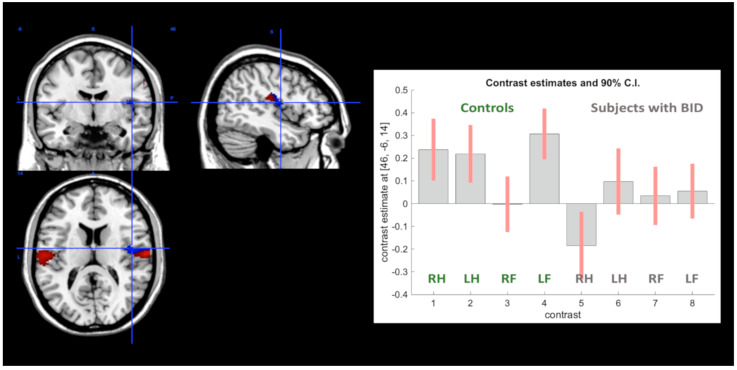
The Group-by-Body Part-by-Side interaction in right area SII for the somatosensory stimulations (voxels in blue)**.** The bar graphs illustrate the contrast estimates—betas—and their 90% confidence intervals of the fMRI response in the right SII (MNI coordinate: x = 46; y = −6; z = 14; *p* = 0.001) in controls and participants with BID during the tactile stimulation of the different body parts. The surrounding areas in red are voxels where the main effect of reduced activation for tactile stimuli in subjects with BID within area SII was observed.

Within the right area SII, there was also a portion showing the crucial Group-by-Body Part by-Side interaction: even though this did not reach a corrected significance level, a *p* = 0.001 level of significance was achieved (x = 46; y = −6; z = 14) (see Figure 2). Consistent with the nature of this effect, the position of this focus within area SII was medial, in agreement with the somatotopy of area SII [56].

**Figure 3:**
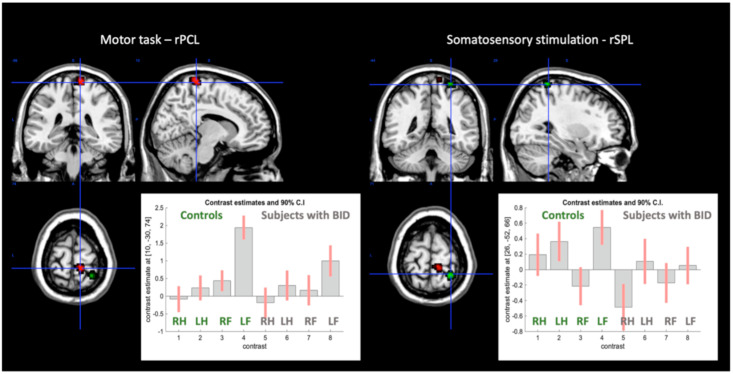
Regional effects in the right paracentral lobule (red area) and in the right superior parietal lobule (green area), regions that in Saetta et al. [28] had altered functional connectivity. The bar-graphs describe the contrast estimates—betas—and their 90% confidence intervals of the fMRI response for each condition in the healthy controls and in the subjects with BID. RH = right hand; LH = left hand; RF = right foot; LF = left foot.

The crucial Group-by-Body Part-by-Side interaction was also present for the Saetta et al. [28] rSPL: this was less active in BID participants specifically for the somatosensory stimulation of the left foot (MNI coordinates: x = 26; y = −52; z = 66, *p* = 0.007) (Figure 3, green region).

##### Motor Execution Task

For the volume FWER cluster wise-corrected analyses, no significant differences in motor execution were found between BID and controls for the movement of any upper or lower limb, nor as a main effect of Group, nor as a Group-by-other factors interactions at a *p* < 0.05 FWER corrected threshold.

In the more lenient ROI oriented analysis of the regions described by Saetta et al. [28], a Group-by-Body Part-by-Side interaction was present in the most dorsal part of the precentral gyrus, corresponding to the right paracentral lobule: this was less active specifically for the left foot movements (x = 10; y = −36; z = 62; *p* = 0.008; x = 10; y = −30; z = 74; *p* = 0.038; Figure 3).

#### 3.2.3. Conjunction Analyses

##### Conjunction of the Effects of the Somatosensory Stimulations

For the tactile stimulation task, there was no area of joint activation for all limbs that was shared by both groups. Indeed, while for the healthy controls there were FWER corrected activations in area SII on both sides of the brain (see Table 4), for the group of individuals with BID there was no brain region showing a conjunction effect unless an uncorrected *p* < 0.05 threshold was imposed on the SPM x = 54; y = −30; y = 20; Z score 2.2.

##### Conjunction of the Effects of Motor Execution

For the motor execution task, there was one region showing a conjunction effect across groups and conditions whereby the response was present and significant for all limbs across groups: the region involved was the supplementary motor area; this survived a correction for multiple comparisons. Once considered at a group-specific level, for the healthy controls the areas of conjunctions involved also ventral premotor cortices bilaterally, and the parietal opercula of both hemispheres. On the other hand, for the subjects with BID, the areas of a joint activation for all motor tasks were restricted to the SMA, the right parietal operculum, and the right ventral premotor cortex.

#### 3.2.4. Comparisons of the Topographical Distribution of the Conjunction Effects

**Figure 4:**
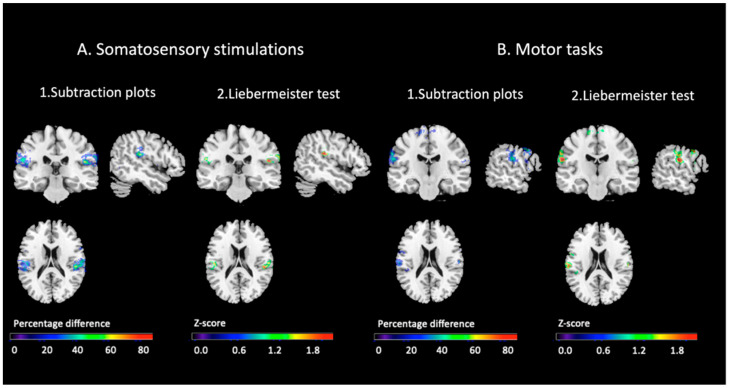
Comparison of the distribution of the conjunction effects for all somatosensory conditions and all motor task conditions. (**A**) Somatosensory task and (**B**) motor task. The Figure shows the subtraction plots between controls and participants with BID in the somatosensory and the motor task (A1 and B1). The percentage overlapping regions of the conjunction of the control group after subtracting the binarized maps of the BID group is illustrated by different colors, coding increasing frequencies from dark blue to red (difference 100%). Only brain regions that were damaged 20% or more frequently in controls than in participants with BID are showed. The figure also shows the result of the Liebermeister test between controls and participants with BID in the somatosensory and motor task (A2 and B2): The color scale illustrates the corresponding Z-score values. Voxels that survived the statistical threshold of *p* < 0.05 (Z-score = 1.64) uncorrected are shown in red.

Comparison of the binarized maps from the two groups and the two tasks led to the observation of a reduced frequency in BID compared to controls of joint activation for all body segments considered in the left parietal operculum corresponding to area SII for both the somatosensory and the motor task, and in the right area SII for the somatosensory task only (see Figure 4). These findings were observed using both the subtraction method to compare the binarized maps of the two groups and the between-group voxel-by-voxel statistical analysis using the Liebermeister test as implemented in the Non-parametric Mapping tool (NPM) included in the MRIcron free software [53].

## 4. Discussion

The notion that each part of our body belongs to us and is thus an intrinsic part of our body identity is one of the most central aspects of our mental life; this is far from being granted and it proves to be liable to distortions. This is shown by the existence of different forms of body representation disorders (e.g., see [19,34,57,58,59,60]). These manifestations may range from body parts misattribution to other people (i.e., somatoparaphrenia, [17]), hatred for body parts (i.e., misoplegia, [61,62]), a reduced experience of embodiment (i.e., pathological embodiment; [63]), feeling that a body part has faded from awareness (i.e., asomatognosia, [59,61]), to different kinds of conflicts between the experienced body (i.e., online body representation, see [64]) and its congenital representation (pre-existing top-down representation, see [36], i.e., offline representation, see [64,65]).

BID is somehow similar to these conditions, as it is characterized by a distressing sense of incongruity between the existent and the desired body structure, something that may lead to the obsessive desire for the amputation of the “exceeding” body segment.

The left-sided bias in the lateralization of the amputation desire in our sample is barely a matter of chance; while not a one hundred percent rule, left-sided cases are prevalent, which constitutes a further similarity with syndromes with acquired brain disorders generally associated with right brain damage, involving, even though not exclusively, the right parietal lobe.

In recent years, evidence of a possible neurological/neuropsychological etiology of the BID has accumulated. However, to date, such evidence remains still somewhat limited [11,18], in particular regarding the presence of task-specific neurofunctional changes that could help to understand the precise nature of the disorder [11,14,23,24,25,26,27,28] (see Table 1).

Recently, we proposed that the malfunctioning of a distributed neural network might explain the BID symptomatology [28]. On the basis of resting-state functional connectivity fMRI data, the network included two regions of the motor and somatosensory system, the right paracentral lobule (rPCL) and the right superior parietal lobule (rSPL): from the present task-based fMRI study we now know that these regions are significantly active, in normal controls, for both somatosensory stimulation and motor activity of the left foot in the case of the paracentral lobule and for somatosensory stimulation for the rSPL.

The task-based fMRI data now show that these regions are less active specifically in subjects with BID for the left limb in a task-dependent manner. The effects in the motor task were limited to a specific reduction in activation of the right paracentral lobule. Groups specific effects for the somatosensory task were more complex and probably more revealing. The rSPL previously implicated in BID [11,14,28] showed a specific reduction in response to somatosensory stimulation of the left foot rather than for a motor task. This was not an isolated finding, as it was accompanied by a similar interaction in the right area SII. Areas SII of both sides had further effects that are worth discussing; we also found that other parts of areas SII had a reduced activation also for the stimulation of the right leg and indeed also for stimulation of the left and right hand. This unexpected finding may surprise and deserves a detailed discussion.

Area SII is well-known to be involved in the high-level processing of tactile stimuli and body representation. In humans, area SII contributes to both tactile [66,67] and pain perception, visual perception, and objects recognition but also to higher-level cognitive functions such as bodily self-awareness (reviewed in [68]).

Contrary to primary somatosensory area S1, area SII contains neurons whose receptive fields are not limited to the opposite side of the body but also responded to ipsilateral or bilateral stimulation of a given body part and indeed for the stimulation of large body segments (e.g., an entire limb) (see review of the non-human primate data in [42]). The left area SII may also be involved in the supramodal representation of one’s own body and may contribute to establishing a highest-order body representation [69].

Taken together these considerations prompted further analysis of the data: with ad-hoc conjunction analyses and a comparison of their distributions across groups, we tested the hypothesis that a smaller degree of convergence to area SII may characterize the brain response and organization of subjects with BID. This was surmised qualitatively by the observation that, while conjunctions for somatosensory stimulation were highly significant in the healthy controls, BID participants evidenced only a modest conjoint response in left SII, and exclusively with an uncorrected *p* < 0.05 threshold. More formally, when we compared the frequency whereby in each voxel a conjunction effect was observed in the two populations, we found that this was significantly more so in the healthy controls compared with the individuals with BID. Interestingly, similar findings emerged also in areas SII for the activations associated with the motor tasks.

Because of the known properties of area SII, where neurons with complex somatosensory receptive fields co-exist in the same narrow space, we propose that a more limited integration of signals from individual limbs within a region with such nature may lead to an eroded body representation. The effects for the left foot in the right superior parietal cortex and in the more medial part of the right area SII add specificity to our observations.

By comparison with the findings of the somatosensory task, the observations from the motor tasks were not as clear-cut. As for the somatosensory task, we found the crucial Group-by-Body Part-by-Side interaction also in the right paracentral lobule and, as for the somatosensory task. On the other hand, the left ventral precentral premotor region, which in Saetta et al. [28] and in Blom and co-workers [23] had less dense grey matter, showed a systematic more limited response for all limbs but only for the somatosensory stimulation conditions. A more limited response of the ventral premotor cortex in a somatosensory task is not totally surprising as the left ventral premotor region is well known to possess multimodal neurons including neurons responding to somatosensory stimuli [70].

Accordingly, as reported previously [27], BID does not seem to be associated with major abnormalities in the functioning of motor networks, and even when these are involved, such as in the case of the ventral premotor cortex, they may be less responsive to their sensory integration function—they were less active in the somatosensory task—rather than for their contribution to motor planning.

It remains to be explored whether the use of more complex motor paradigms, perhaps tapping into forms of explicit awareness about acting and sense of agency, will determine the emergence of differences in brain motor organizations in subjects with BID. For the time being there is neither behavioral nor physiological evidence along these lines.

In returning to the “somatosensory findings” our observations confirm and expand what was described by McGeoch and collaborators who found a reduction in MEG activity of the right superior parietal cortex (rSPC) 40–140 ms after the tactile stimulation of the affected limb in BID [11]. The observed anomalies were selective for the tactile stimulation of the affected limb (i.e., the right leg in two subjects, and the left leg in the other two) and restricted to the rSPC since the other regions tested, such as the primary somatosensory cortex (SI), but also the IPL, M1, the insula, the premotor cortex, and the precuneus, did not show any BID-specific response [11].

All that said, it remains to be discussed how our imaging finding may justify the main symptoms of BID and why a reduced functionality within somatosensory areas such as, for example, both areas SII does not lead to clinically visible somatosensory deficits.

Indeed, the subjects with BID included in our study did not show tactile deficits in the clinical evaluation: they could correctly perceive light touches administered on both hands and feet (standardized clinical evaluation; [44]) and they did not report altered tactile sensations on the affected limb. Abnormal sensory experiences such as paraesthesia and hypoesthesia have been reported in participants with BID [12] using questionnaires. Moreover, individuals with BID reported differences in sensation between the affected and unaffected limb in the direction of both increased (15%) and reduced sensitivity to touch (14%) [13]. However, when tested systematically, such individuals do not have more tactile perception deficits on the part of the body for which they feel a desire for amputation [15].

These considerations illustrate how the interpretation of the reported hypo-activations is still open to discussion. Could these hypo-activations be due to attentional phenomena? There are at least three arguments that militate against this hypothesis. On the one hand, the obsessive rumination on the desire of being amputated may allow one to expect increased responses in somatotopically specific brain regions rather than hypo-activations. This was not the case. Second, an attentional phenomenon cannot easily explain the reduced activations in area SII for stimulations of all body segments. Third, the brain response reductions in area SII were specific for the somatosensory stimulations while no similar differences were observed for the motor tasks. In addition, the functional connectivity abnormalities described in Saetta et al. [28] are in regions that here we demonstrate are specifically active in healthy controls for tactile stimulations or actions of the left foot.

Overall, while our results indicate a global alteration of the brain networks involved in multisensory integrations and body representation, it remains unclear how the observed brain anomalies in the processing of somatosensory stimuli are linked with the feeling of over-completeness and the consequent desire for amputation of a specific limb or paralysis on one side of the body. The conceptual riddle comes from the need to interpret what is essentially a “productive symptom” (the obsessive desire for amputation) with a reduction in function. Clearly, the reduced activity described cannot by itself account for the symptom if we make the analogy with an anatomical lesion. Yet it may be the prerequisite for its manifestation much as acquired brain lesions are for similar disorders of body representation as in somatoparaphrenia or for altered feelings of embodiment. These manifestations are all characterized by a delusional, productive, component in the context of the most extreme form of a local reduction in function such as that of a definitive brain lesion.

Taken together, these previous findings and those reported here help us to decide on whether one of the scenarios, of the four envisaged in our introduction, may better capture the dysfunctional anatomy of BID: contrary to our initial predilection for a “Sensorimotor integration” scenario C, none of the scenarios that we anticipated materialized in full: indeed, here we found a normal elementary somatosensory discrimination and normal elementary motor functions in face of a reduced response of area SII and left ventral premotor cortex for stimulation of all limbs but not for the motor task; this was accompanied by some left-leg-specific reduction in response of somatosensory regions that somatotopically correspond to the left leg and a minor involvement of the right paracentral lobule for the left foot movements. This last finding may still depend on the reduced response of somatosensory neurons of area 3a at the border with the primary motor cortex, two regions that we cannot resolve spatially here. Taken together all the above, a modified **Higher-order somatosensory scenario B** may be better justified by our data, where the high-orderliness is not represented solely by the anatomical nature of the brain regions involved, rather by what we presume to be a lack of integration of the signals coming from one limb into a more general body image logic, as also testified by the previous evidence of reduced functional connectivity [28].

## 5. Limitations of the Study

There are three limitations that we wish to acknowledge here.

First, sample sizes: While it is true that the present study represents the larger homogeneous sample of BID individuals with the same desire for amputation of their left leg studied with a task-based fMRI paradigm, the sample size of our study remains relatively small in absolute terms. This should be kept in mind when reasoning about the generalizability of the present findings. In any event, a random effect analysis and conservative thresholds were used to maximize the chance that our task-based fMRI observations could be replicated in the future.

Second: Independence of the present observations from previous reports (e.g., [28]). The ROIs used in our analyses were derived by a sample of 16 individuals with BID [28], eight of which are included in the present paper. Therefore, even if the ROIs are not entirely independent from the data reported in Saetta et al. [28], the two samples overlap only in part. Notably, the nature of the signal sampled by the new study is entirely different from the one described in Saetta et al. [28] as it was derived from task-based paradigms rather than resting-state fluctuations of the BOLD signal. At the very least, the present study may be seen as an extension of Saetta et al. [28]. Yet, the different nature of the signals described and the findings are novel and well beyond Saetta et al. [28] report.

Third: The distinction between nature or nurture in BID. Concerning the nature versus nurture dilemma (see also [2,71]), our results do not allow us to state whether the observed cerebral alterations, mostly seen for somatosensory functions, are the cause of BID or whether, on the contrary, it is the typical clinical symptomatology of BID, which afflicts subjects from early childhood, to determine the brain differences observed. In principle, the observation of brain alterations could indicate that their presence determines or triggers the disorder. However, the opposite hypothesis cannot be excluded, namely that the disease may have somehow caused these brain alterations. As Sedda and Bottini discussed [2], only genetic studies may have the potential to provide causal explanations to the disorder.

## 6. Conclusions

Inevitably, the evidence provided here cannot but represent a further step towards a more comprehensive interpretation of BID. Having discovered that BID is associated with perturbed physiology of brain networks involved in body representation and more so for somatosensory rather than motor functions, it remains to be discovered how the malfunctional operations of these networks feed into the brain systems that normally contribute to the feeling that our body is complete as it is to generate a perturbated one.

Despite all the remaining unclear aspects of BID, on a practical clinical level, it is relevant to have discovered and confirmed that this condition is associated with the malfunctions of easily accessible cortical regions involved in body representation. It may be worth further experimenting with a more systematic approach, with the neuromodulation of these regions. Techniques such as, for example, transcranial magnetic stimulation (TMS, see [72]) or transcranial direct current stimulation (tDCS) may be prime candidates to be exploited for the alleviation of the dysphoria associated with amputation desires or their variants.

## Figures and Tables

**Table 1:** List of studies that have investigated the neurofunctional bases of BID using neuroimaging techniques.

Author(s) ^1^	Year	Sample Size		Side			Body Part	Technique	Analysis	Structural/Functional Abnormalities
		BID	C	L	R	B				
McGeoch et al. [11]	2011	4	4	1	2	1	Leg	MEG	ROIs-based	Reduced activity in the rSPL.
Hilti et al. [14]	2013	13 *^,^^	13	8	2	3	Leg	MRI—surface-based morphometry	ROIs-based	Reduced thickness: RSPL. Increased thickness: rCS, rIPL. Reduced cortical surface area: rIPL, rSI, rSII, r anterior IC. Increased cortical surface area: lIPL, lSII.
van Dijk et al. [27]	2013	5	10	2	3	0	Leg	Task-based fMRI	Whole brain	Tactile task: reduced activation in the PMCd and PMCv for the affected leg; Heightened responsivity in a large somatosensory network regardless of which leg was stimulated. Motor task: no differences.
Blom et al. [23]	2016	8	24	2	3	3 ^#^	Leg	MRI (VBM)	Whole brain and SVC in predefined ROIs	Reduced grey matter concentration: ldPMC, lvPMC; Increased grey matter concentration: lCerebellum.
Hänggi et al. [24]	2016	13 *	13	8	2	3	Leg	MRI—FIRST	Subcortical regions	Thinning (hypotrophy): bilateral dorsomedial putamen, left ventromedial caudate, left medial pallidum. Thickening (hypertrophy): bilateral lateral pallidum; left frontolateral thalamus.
Hänggi et al. [25]	2017	13 *	13	8	2	3	Leg	MRI; DTI; rsfMRI	Whole brain connectome; network analyses in predefined nodes	Hyperconnected nodes: Connectome analysis: Paracentral lobule, SMA, Postcentral gyrus, BG, Cerebellum. Network analyses: SPL, SI, SII, PMC, BG, Thalamus, IC.
Oddo et al. [26]	2018	12	11	9	1	2	Leg	fMRI	Machine-learning—whole brain	Brain activity evocated by viewing images of the own virtually amputated body predicts BID. bSPL, IPL, caudate nucleus and occipital regions are among the highest predictive regions.
Saetta et al. [28]	2020	16 **	16	16	0	0	Leg	RsfMRI—MRI	Whole brain rsfMRI and ROIs-based MRI (VBM)	rPCL; rSPL, lSTG, and lIFGOrb, were less functionally connected to the rest of the brain. lPMC and rSPL, and lIFGOrb were atrophic.

^1^ fMRI studies that used active tasks (i.e., tactile, motor, or cognitive tasks) are highlighted in light grey. C = Controls; L = left; R = right; B = bilateral; MEG = magnetoencephalography; MRI: magnetic resonance imaging; fMRI = functional magnetic resonance imaging; rsfMRI = resting state fMRI; FIRST = FMRIB’s Integrated Registration and Segmentation Tool (http://fsl.fmrib.ox.ac.uk/fsl/fslwiki/FIRST, accessed on 16 September 2021). l = left hemisphere; r = right hemisphere; b = bilateral; d = dorsal; v = ventral. SVC = small volume correction; VBM = Voxel-based morphometry; SPL = Superior parietal lobe; CS = Central sulcus; IPL = Inferior parietal lobe; SI = Primary Somatosensory Cortex; SII = Secondary Somatosensory Cortex; IC = Insular Cortex; PMC = Premotor cortex; SMA = Supplementary motor area; BG = Basal ganglia; STG = superior temporal gyrus; inferior orbitofrontal gyrus (IFGOrb). PCL = paracentral lobule. Notes about samples: * BID participants are the same in the study of Hilti et al. [14], Hänggi et al. [24] and Hänggi et al. [25] (*n* = 13); ** eight of the 16 subjects included in Saetta et al. [28] participated in the study of Hilti et al. [14], and eight are included in the present study. ^ only 13 of the 15 recruited participants met the inclusion criteria for the MRI study. ^#^ 1 subject desired both leg paralysis, 1 subject desired bilateral upper legs amputation, 1 subject desired lower back paralysis.

**Table 2:** Demography and clinical features of participants with BID. * Range 1–6.

Participants with BID	Demographic Features		BID Features		Mean Scores on Zurich Xenomelia Scale *			
	Age	Education	Limb	Side	Pure Amputation Desire	Erotic Attraction	Pretending Behaviour	Total Scale Scores
P1	42	13	leg	left	4.50	4.25	5.00	4.58
P2	36	13	leg	left	4.50	4.25	5.00	4.58
P3	36	13	leg	left	5.25	3.25	3.00	3.83
P4	48	18	leg	left	6.00	3.50	4.00	4.50
P5	34	18	leg	left	5.25	2.75	3.25	3.75
P6	37	18	leg	left	6.00	6.00	4.75	5.58
P7	41	13	leg	left	5.75	4.50	5.00	5.08
P8	39	18	leg	left	6.00	4.25	5.50	5.25
P9	64	13	leg	left	6.00	5.50	4.50	5.33
P10	38	14	leg	left	6.00	3.75	3.00	4.25

**Table 3:** Between-group comparison: tactile stimulation task. MNI coordinates of the brain regions were significantly less active in subjects with BID than in healthy subjects during the tactile stimulation of the body parts (controls > participants with BID). Brain region (R = right hemisphere; L = left hemisphere), cluster size (k = number of voxels), cluster-wise FWER-corrected *p*-value and uncorrected *p*-value, voxel-wise (peak level) Z-score and Montreal Neurological Institute (MNI) coordinates are reported. ^#^ Statistical threshold *p* < 0.05 whole-brain FWER-corrected voxelwise (peak level). BA = Brodmann Areas; corr = corrected. Cluster 3 is reported because it corresponds to the superior parietal lobule of reduced connectivity and grey matter density of Saetta et al. [28]. The left precentral peak of cluster 2 includes the precentral area of reduced grey matter density reported in Saetta et al. [28]. ** brain regions identified by the Neuromorphometrics and the HarvardOxford atlas.

Brain Region (BA)	Cluster			Peak			
	K	P_FWER-corr_	P_uncorrected_	Z-Score	MNI Coordinates		
					x	y	z
***Cluster 1***	***765***	***0.005***	***0.002***				
R Postcentral gyrus				5.06 ^#^	62	−14	18
R Postcentral gyrus (43)				3.65	62	−10	38
R Supramarginal gyrus (2)				4.52 ^#^	66	−22	34
***Cluster 2***	***1625***	***<0.001***	***<0.001***				
L Supramarginal gyrus (1)				4.72 ^#^	−62	−22	36
L Postcentral gyrus				4.66 ^#^	−60	−14	18
L Precentral gyrus (6)				4.18	−50	2	22
***Cluster 3***	***174***	***0.254***	***0.090***				
R Postcentral gyrus—SPL **				3.51	26	−46	64
R Inf. parietal gyrus (3)				3.46	30	−42	54

**Table 4:** Conjunction of the effects of the motor and somatosensory tasks. MNI coordinates of the brain regions commonly activated for the four conditions of the motor tasks (1) and the four conditions of the somatosensory tasks (2). Brain regions (R = right hemisphere; L = left hemisphere), cluster size (k = number of voxels), FWER-corrected *p*-value and uncorrected *p*-value, peak Z-score and Montreal Neurological Institute (MNI) coordinates are reported. ^#^ Statistical threshold *p* < 0.05 whole-brain FWER-corrected voxelwise. BA = Brodmann areas; corr = corrected. ** labeled as no region in the AAL template and identified by the Neuromorphometrics and the HarvardOxford atlas.

Brain Regions (BA)	Cluster			Peak			
	K	P_FWER-corr_	P_uncorrected_	Z-Score	MNI Coordinates		
					x	y	z
**(1) MOTOR TASKS**							
**(a) Conjunction Controls and BID**							
***Cluster 1***	**468**	**0.021**	**0.006**				
R SMA (6)				4.52 ^#^	6	−4	60
L SMA (6)				3.70	−8	−12	64
**(b) Conjunction Controls**							
***Cluster 1***	**1600**	**<0.001**	**<0.001**				
R SMA				7.0 ^#^	2	−4	58
R Mid. Cingulum (24)				3.29	10	6	36
***Cluster 2***	**845**	**0.002**	**<0.001**				
L Precentral gyrus (6)				5.27 ^#^	−60	6	28
L Precentral gyrus (6)				4.59 ^#^	−46	−6	54
L Rolandic opercular gyrus				4.33	−48	2	10
***Cluster 3***	**432**	**0.027**	**0.007**				
R Supramarginal gyrus				4.55 ^#^	62	−16	22
R Supramarginal gyrus				4.05	54	−26	34
R Superior temporal gyrus				3.12	58	−34	24
***Cluster 4***	**297**	**0.079**	**0.022**				
L Supramarginal gyrus				4.49 ^#^	−56	−22	24
***Cluster 5***	**463**	**0.022**	**0.006**				
R Insula				3.93	44	2	6
R Rolandic opercular gyrus				3.83	54	8	14
R Putamen				3.62	28	−2	0
**(c) Conjunction BID**							
***Cluster 1***	**470**	**0.021**	**0.005**				
R SMA (6)				4.52 ^#^	6	−4	60
L SMA (6)				3.70	−8	−12	64
***Cluster 2***	**461**	**0.022**	**0.006**				
R Rolandic opercular gyrus				3.82	60	−18	16
R Supramarginal gyrus**				3.73	48	−26	28
R Supramarginal gyrus				3.68	56	−24	36
**(2) SOMATOSENSORY TASKS**							
**(a) Conjunctions in Controls and BID**							
	No areas of shared activations						
**(b) Conjunctions in Controls**							
***Cluster 1***	**956**	**0.002**	**0.001**				
L Postcentral gyrus				5.15 ^#^	−54	−18	18
L Supramarginal gyrus				4.97 ^#^	−54	−26	22
L Supramarginal gyrus (43)				4.29	−60	−20	36
***Cluster 2***	**735**	**0.006**	**0.002**				
R Rolandic opercular gyrus				4.93 ^#^	50	−28	22
**(c) Conjunctions in BID**							
	No areas of shared activations						

## Data Availability

The data presented in this study are available on request from the corresponding author. The data are not publicly available due to privacy and ethical concerns.

## Supplementary Materials

The following are available online at https://www.mdpi.com/article/10.3390/brainsci11091248/s1, Figure S1: Tactile stimulation task. Brain activation patterns for participants with BID (left column) and controls (right column). The main effect of touch for hands and feet (i.e., hands > rest; feet > rest) were thresholded at *p* < 0.05 corrected for multiple comparisons (FWER-corrected) and render on the left and right hemisphere. The color scales indicate the significance of the SPMs [Z] maps. Figure S2: Tactile stimulation: comparison between hands and feet. Brain activation patterns for participants with BID and controls during the tactile stimulation of hands and feet. In blue are reported activations for the contrast feet > hands and in red activation for the contrast hands > feet. All activations were thresholded at *p* < 0.05 corrected for multiple comparisons (FWER-corrected) and render on an MNI template. Figure S3: Motor Execution task. Brain activation patterns for participants with BID (left column) and controls (right column). The main effect of the hands and feet movement (i.e., motor execution hands > rest; motor execution feet > rest) were thresholded at *p* < 0.05 corrected for multiple comparisons (FWER-corrected) and render on the right and left hemisphere. The color scales indicate the significance of the SPMs [Z] maps. Table S1: Tactile stimulation task. MNI coordinates of the brain regions active in subjects with BID and controls during the tactile stimulation of the hands and feet. Brain region (R = right hemisphere; L = left hemisphere), cluster size (k = number of voxels), cluster-wise FWER-corrected *p*-value and uncorrected *p*-value, voxel-wise (peak level) Z-score and Montreal Neurological Institute (MNI) coordinate are reported. Only voxels that survived the cluster-wise *p* < 0.05 FWER corrected threshold are reported. ^#^ Statistical threshold *p* < 0.05 whole-brain FWER-corrected voxelwise. The local maxima of significant clusters are reported in MNI coordinates. BA = Brodmann Areas; corr = corrected. ** labelled as no region in the AAL template and identified using the Neuromorphometrics and the HarvardOxford atlas. Table S2: Motor execution task. MNI coordinates of the brain regions active in subjects with controls and individuals with BID during the movement of hands and feet. Brain region (R = right hemisphere; L = left hemisphere), cluster size (k = number of voxels), cluster-wise FWER-corrected *p*-value and uncorrected *p*-value, voxel-wise Z-score and Montreal Neurological Institute (MNI) coordinate are reported. The voxel-wise threshold applied to the statistical maps before the cluster-wise correction was *p* < 0.05 FWER corrected level. The local maxima of significant clusters are reported in MNI coordinates. BA = Brodmann areas; corr = corrected. ** labelled as no region in the AAL template and identified using the Neuromorphometrics and the HarvardOxford atlas.
