# Supplementary material for: Brain Abnormalities in Individuals with a Desire for a Healthy Limb Amputation: Somatosensory, Motoric or Both? A Task-Based fMRI Verdict
Source: Brain Sci. 2021 Sep 21;11(9):1248. doi: 10.3390/brainsci11091248 (PMC8468102; doi:10.3390/brainsci11091248)
Supplement: Supplementary file 1 [file brainsci-11-01248-s001.zip › brainsci-1338792-supplementary/Supplemetary figures and tables/Table S1.pdf]

Table S1: Tactile stimulation task. MNI coordinates of the brain regions active in subjects with BID and controls during the tactile stimulation of the hands and feet. Brain region (R = right hemisphere; L = left hemisphere), cluster size (k = number of voxels), cluster-wise FWER-corrected p-value and uncorrected p-value, voxel-wise (peak level) Z-score and Montreal Neurological Institute (MNI) coordinate are reported. Only voxels that survived the cluster-wise p<0.05 FWER corrected threshold are reported. #Statistical threshold p < 0.05 whole-brain FWER-corrected voxelwise. The local maxima of significant clusters are reported in MNI coordinates. BA= Brodmann Areas; corr = corrected. **labelled as no region in the AAL template and identified using the Neuromorphometrics and the HarvardOxford atlas.

Brain region (BA)	Cluster			Peak			
	K	P FWER-corr	P uncorrected	Z-score	MNI coordinates		
					x	y	z
**1. CONTROLS**							
**A. Right hand**							
Cluster 1	***9948***	***<0.001***	***<0.001***				
L Postcentral gyrus (3				Inf#	-48	-26	56
L Rolandic opercular gyrus				Inf#	-50	-20	18
L SMA				5.93#	-6	-8	50
Cluster 2	***754***	***0.005***	***0.002***				
R Cerebellum				6.97#	22	-52	-26
Cluster 3	***2646***	***<0.001***	***<0.001***				
R Rolandic opercular gyrus				6.23#	58	-18	20
R Supramarginal gyrus (43)				5.32#	62	-20	36
R Inf. parietal gyrus (2)				4.51#	50	-34	54
Cluster 4	***482***	***0.028***	***0.009***				
R Mid. temporal gyrus (37)				5.09#	54	-58	0
Cluster 5	***801***	***0.004***	***0.001***				
R Insula				4.52#	42	0	10
R Insula				4.48#	44	2	0
R Rolandic opercular gyrus				3.95	54	10	4
**B. Left hand**							
Cluster 1	***7123***	***<0.001***	***<0.001***				
R Postcentral gyrus (4)				Inf#	40	-24	54
R Rolandic opercular gyrus				Inf#	52	-18	18
R Insula				4.87#	42	-2	8
Cluster 2	***826***	***0.004***	***0.001***				
L Cerebellum				Inf#	-20	-52	-26
Cluster 3	***1972***	***<0.001***	***<0.001***				
L Supramarginal gyrus (3)				5.52#	-58	-22	40
L Postcentral gyrus				5.31#	-52	-18	16
L Supramarginal gyrus				4.97#	-54	-26	22
**C. Right foot**							
Cluster 1	***962***	***0.002***	***0.001***				
L Precuneus (5)				6.59#	-12	-44	64
L Paracentral lobule				6.01#	-6	-36	62

L Paracentral lobule (6)				4.51#	-8	-14	68
Cluster 2	***1801***	***<0.001***	***<0.001***				
L Sup. temporal gyrus				6.13#	-50	-28	20
L Heschl gyrus				5.53#	-34	-22	14
L Supramarginal gyrus (2)				5.34#	-60	-24	40
Cluster 3	***741***	***0.006***	***0.002***				
R Rolandic opercular gyrus				4.94#	48	-28	22
**C. Left foot**							
Cluster 1	***1387***	***<0.001***	***<0.001***				
R Postcentral gyrus (5)				Inf#	14	-42	68
Cluster 2	***3538***	***<0.001***	***<0.001***				
R Sup. temporal gyrus				7.74#	50	-30	20
R Rolandic opercular gyrus				7.07#	56	-20	18
R Postcentral gyrus (43)				4.96#	62	-16	36
Cluster 3	***2021***	***<0.001***	***<0.001***				
L Supramarginal gyrus				6.48#	-52	-26	20
L Supramarginal gyrus (43)				4.29#	-60	-20	36
L Rolandic opercular gyrus				4.17	-44	-4	16
**2. BID**							
**A. Right hand**							
Cluster 1	***2671***	***<0.001***	***<0.001***				
L Postcentral gyrus (3)				Inf#	-38	-24	52
Cluster 2	***468***	***0.030***	***0.009***				
R Cerebellum				5.88#	24	-52	-26
**B. Left hand**							
Cluster 1	***2581***	***<0.001***	***<0.001***				
R Postcentral gyrus (4)				Inf#	38	-26	56
R Precentral gyrus (6)				7.53#	38	-14	62
**C. Right foot**							
Cluster 1	***1437***	***<0.001***	***<0.001***				
L Paracentral lobule				Inf#	-6	-32	62
L Precuneus (4)				Inf#	-14	-40	66
Cluster 2	***797***	***0.004***	***0.001***				
L Sup. temporal gyrus				4.90#	-46	-30	18
L Parietal operculum** (41)				4.56#	-40	-38	22
L Parietal operculum**				3.45	-26	-22	20
**C. Left foot**							
**Cluster 1**	**1017**	***<0.001***	***<0.001***				
R Postcentral gyrus (4)				7.71#	12	-40	66
R Paracentral lobule (4)				7.09#	6	-30	62
**Cluster 2**	**1096**	**0.001**	***<0.001***				
R Sup. temporal gyrus				6.63#	48	-30	22
R Insula**				4.03	32	-24	16
