# Supplementary material for: Brain Abnormalities in Individuals with a Desire for a Healthy Limb Amputation: Somatosensory, Motoric or Both? A Task-Based fMRI Verdict
Source: Brain Sci. 2021 Sep 21;11(9):1248. doi: 10.3390/brainsci11091248 (PMC8468102; doi:10.3390/brainsci11091248)
Supplement: Supplementary file 1 [file brainsci-11-01248-s001.zip › brainsci-1338792-supplementary/Supplemetary figures and tables/Table S2.pdf]

Table S2: ***Motor execution task.*** MNI coordinates of the brain regions active in subjects with controls and individuals with BID during the movement of hands and feet. Brain region (R = right hemisphere; L = left hemisphere), cluster size (k = number of voxels), cluster-wise FWER-corrected p-value and uncorrected p-value, voxel-wise (peak level) Z-score and Montreal Neurological Institute (MNI) coordinate are reported. Only voxels that survived the cluster-wise p<0.05 FWER corrected threshold are reported. #Statistical threshold p < 0.05 whole-brain FWER-corrected voxelwise. The local maxima of significant clusters are reported in MNI coordinates. BA= Brodmann Areas; corr = corrected. **labelled as no region in the AAL template and identified using the Neuromorphometrics or the HarvardOxford atlas.

Brain region (BA)	cluster- level			voxel-level			
					MNI coordinates		
	K_E	$P_{FWER\ corr}$	$P_{uncorrected}$	Z-score	x	y	z
**1. CONTROLS**							
**A. Right hand**							
Cluster 1	***16222***	***<0.001***	***<0.001***				
L Postcentral gyrus (3)				Inf#	-36	-22	52
L Precentral gyrus (6)				Inf#	-32	-16	60
L SMA (6)				7.56#	-4	-6	54
Cluster 2	***3862***	***<0.001***	***<0.001***				
R Cerebellum				Inf#	12	-54	-18
R Cerebellum				6.92#	14	-64	-48
L Cerebellum				5.31#	-28	-54	-28
Cluster 3	***3065***	***<0.001***	***<0.001***				
R Supramarginal gyrus (2)				5.82#	44	-32	44
R Supramarginal gyrus				4.55#	62	-16	22
R Sup. parietal gyrus (7)				4.22	32	-56	56
Cluster 4	***643***	***0.006***	***0.002***				
R Inf. temporal gyrus (21)				4.69#	54	-52	-4
R Mid temporal gyrus (21)**				4.43	48	-50	2
**B. Left Hand**							
Cluster 1	***14434***	***<0.001***	***<0.001***				
R Precentral gyrus (4)				Inf#	38	-18	54
R SMA (6)				7.46#	2	-4	56
L Precentral gyrus (6)				6.99#	-32	-12	58
Cluster 2	***2963***	***<0.001***	***<0.001***				
L Cerebellum				Inf#	-16	-54	-22
R Cerebellum				5.66#	24	-54	-24
Cluster 3	***1604***	***<0.001***	***<0.001***				
R Pallidum				5.53#	28	-2	-4
R Precentral gyrus (6)				5.15#	60	8	30
R Thalamus				4.54#	16	-20	4
**C. Right foot**							
**Cluster 1**	**26312**	***<0.001***	***<0.001***				

L Paracentral lobule (4)				Inf#	-6	-28	62
L Paracentral lobule (4)				Inf#	-8	-20	68
L Precuneus				Inf#	-6	-38	62
**Cluster 2**	**868**	**0.001**	***<0.001***				
R Vermis				6.58#	4	-48	-18
R Cerebellum**				6.52#	12	-42	-26
L Cerebellum				3.44	-8	-32	-16
**C. Right foot**							
**Cluster 1**	**26312**	***<0.001***	***<0.001***				
L Paracentral lobule (4)				Inf#	-6	-28	62
L Paracentral lobule (4)				Inf#	-8	-20	68
L Precuneus				Inf#	-6	-38	62
**Cluster 2**	**868**	**0.001**	***<0.001***				
R Vermis				6.58#	4	-48	-18
R Cerebellum**				6.52#	12	-42	-26
L Cerebellum				3.44	-8	-32	-16
**2. BID**							
**A. Right hand**							
Cluster 1	***22527***	***<0.001***	***<0.001***				
L Precentral gyrus (6)				Inf#	-32	-14	60
L Postcentral gyrus (4)				Inf#	-36	-24	54
L Inf. parietal gyrus (3)				7.35#	-48	-24	48
Cluster 2	***5753***	***<0.001***	***<0.001***				
R Cerebellum (19)				7.41#	12	-54	-18
R Cerebellum (37)				7.35#	24	-52	-24
L Cerebellum (19)				5.37#	-18	-64	-26
Cluster 3	***5214***	***<0.001***	***<0.001***				
R Supramarginal gyrus (3)				6.04#	52	-24	40
R Mid temporal gyrus**				4.75#	46	-30	-8
R Sup. parietal gyrus (7)				4.39	24	-68	50
**B. Left hand**							
Cluster 1	***17900***	***<0.001***	***<0.001***				
R Precentral gyrus (4)				Inf#	36	-18	54
R Postcentral gyrus (3)				Inf#	36	-32	58
R Postcentral gyrus (3)				Inf#	52	-20	42
Cluster 2	***3899***	***<0.001***	***<0.001***				
L Cerebellum (37)				Inf#	-18	-54	-24
L Cerebellum				5.49#	-10	-68	-44
R Cerebellum				5.45#	26	-56	-26
Cluster 3	***4763***	***<0.001***	***<0.001***				
L Precentral gyrus (6)				5.74#	-56	4	30
L Postcentral gyrus				5.56#	-54	-18	30
L Supramarginal gyrus (2)				5.41#	-48	-30	36
**C. Right foot**							

Cluster 1	***3901***	***<0.001***	***<0.001***				
L Paracentral lobule (4)				Inf#	-4	-28	62
L Paracentral lobule (6)				6.74#	-8	-14	66
R SMA				5.38#	2	-4	62
Cluster 2	***444***	***0.025***	***0.007***				
R Cerebellum**				5.38#	12	-42	-26
R Vermis				4.50#	6	-48	-18
Cluster 3	***2701***	***<0.001***	***<0.001***				
R Sup. temporal pole (38)				5.31#	60	8	-2
R Insula				4.33	48	6	4
R Rolandic opercular gyrus (22)				4.24	62	-18	16
Cluster 4	***5110***	***<0.001***	***<0.001***				
L Putamen				4.63#	-26	-10	8
L Sup. temporal gyrus (41)				4.60#	-50	-32	18
L Thalamus				4.50#	-18	-18	6
**D. Left foot**							
Cluster 1	***1777***	***<0.001***	***<0.001***				
R Paracentral lobule (4)				7.00#	6	-28	64
Cluster 2	***1049***	***<0.001***	***<0.001***				
R Supramarginal gyrus				4.91#	50	-30	24
Cluster 3	***337***	***0.057***	***0.015***				
R Sup. temporal pole (38)				4.30	60	8	-2
Custer 4	***225***	***0.145***	***0.041***				
R Caudate				4.20	16	-8	20
Cluster 5	***576***	***0.010***	***0.003***				
R Pallidum				3.94	26	0	0
R Thalamus**				3.35	8	0	6
