# Supplementary figures and images for: Brain Abnormalities in Individuals with a Desire for a Healthy Limb Amputation: Somatosensory, Motoric or Both? A Task-Based fMRI Verdict
Source: Brain Sci. 2021 Sep 21;11(9):1248. doi: 10.3390/brainsci11091248 (PMC8468102; doi:10.3390/brainsci11091248)

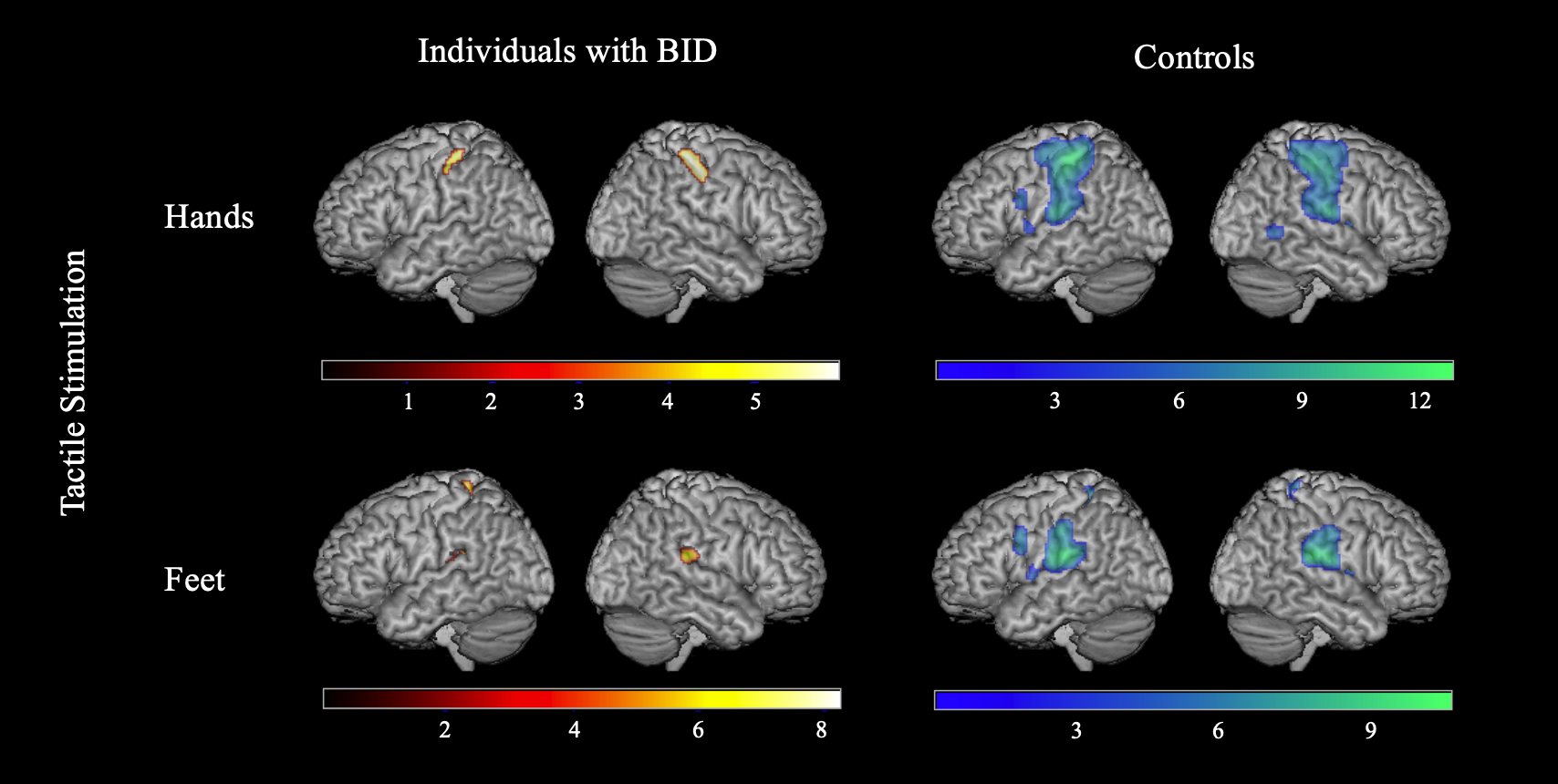

Supplement: Supplementary file 1 [file brainsci-11-01248-s001.zip › brainsci-1338792-supplementary/Supplemetary figures and tables/Figure S1.tiff]

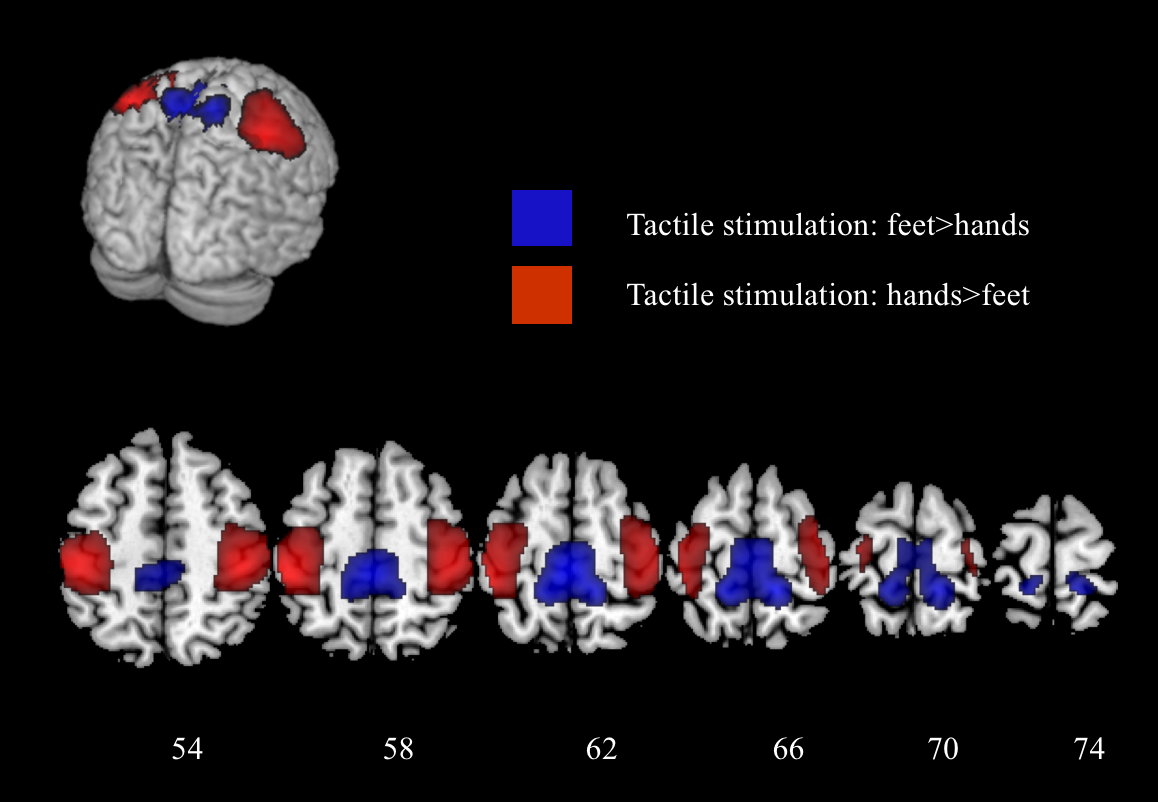

Supplement: Supplementary file 1 [file brainsci-11-01248-s001.zip › brainsci-1338792-supplementary/Supplemetary figures and tables/Figure S2.tiff]

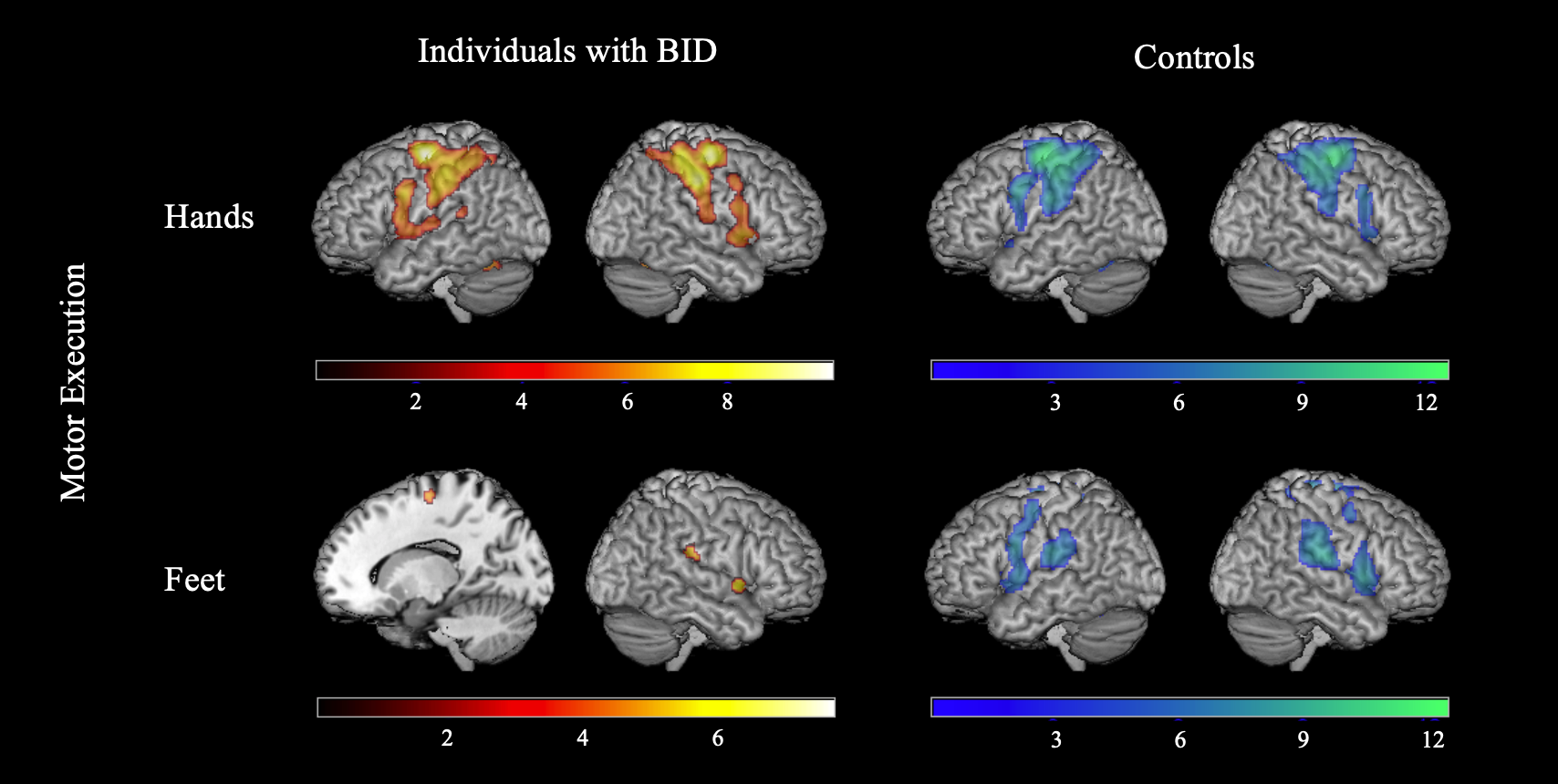

Supplement: Supplementary file 1 [file brainsci-11-01248-s001.zip › brainsci-1338792-supplementary/Supplemetary figures and tables/Figure S3.tiff]
